# Mental Institutions and the War

**Published:** 1914-10-31

**Authors:** 


### MENTAL INSTITUTIONS AND THE WAR.

Hospitals for the treatment of wounded soldiers are not the only medical institutions whose work has been much affected by the war. That every national upheaval is associated with emotional crises is so obvious that it almost amounts to a truism. It is, however, interesting to note that among the secondary effects of this emotional stress is the increase of cases of certifiable insanity. Statistics in regard to the definite dependence of this increase upon emotional stress due to the occurrence of hostilities are apt to be misleading; for instance, economic conditions may lead to patients of the well-to-do classes being sent to mental institutions—their relatives finding that home-nursing of such cases is too expensive at a time when expenditure is being cut down in every possible direction. Apart, however, from the increase in the "asylum population" due to other causes, there is no doubt that many cases of mental breakdown can be traced directly to the emotional stress which war entails. The histories of individual cases are of chief value in proving this. These tend to indicate, as we might expect, that it is, for the most part, the unstable who give way under the strain; in other words, those who have had previous attacks of insanity provide the greater proportion of the increase in the number of admissions. It may, of course, be argued that many of these cases had arrived at a period when recurrence of the attacks would inevitably have taken place; and that the fact that their delusions take a particular colouring is in accordance with what has been observed for many years past, as, for example, in the ideas of demoniacal possession during the earlier period of the Christian era, and the assertion of maleficent powers as witches during the sixteenth and seventeenth centuries. Even if this be granted, however, there still remain numerous cases wherein the onset or the recurrence of an attack of insanity can be definitely traced to this particular emotional stress.

Examples are easy to quote. A man and his wife were in Paris just after the outbreak of hostilities: the husband was arrested as a spy, and ran the risk of being shot. Unable to exculpate himself he became depressed, and attempted suicide: the wife, who had been insane some years previously, became acutely maniacal. A German Jew, whose relatives were in Germany when war was declared, became intensely depressed, feared that he was going to be persecuted by the English and perhaps to be shot. He exhibited marked negativism, and food was only administered with difficulty. His history also included the fact of a previous mental breakdown. Another patient became depressed, and refused food because she believed that she ought not to take nourishment whilst so many poor refugees and others were starving. In this instance the patient had not previously been insane. Yet another patient, who had had several attacks in past years, became convinced that Lord Kitchener had put him in command of a regiment, and he has steadily become more and more excitable. The cases cited will suffice to show that wars and rumours of wars have a definite effect in upsetting the mental processes. When it is realised that they are only a few typical cases, some idea may be formed of the extent of yet another evil among the many which result from warfare.

Again, if the emotional stress proves such a potent factor among the civil population—and these removed by distance from the scene of combat—how much greater must the strain be upon those who are actually participants in the fighting, or who are, like the inhabitants of the invaded districts, exposed to the excesses of a necessarily war-hardened soldiery? Those who are qualified to speak on such matters—as a very remarkable passage in the article called "With a British Field Ambulance in August," written by an Army Medical Officer at the Front, in The Hospital, October 17, p. 63, conclusively proved—leave us in no doubt as to the baneful influence which is exercised by such sights and shocks upon those whose nervous systems are at all unstable. Pinel quotes cases whom the horrors of the French Revolution had made insane, and who came under his care at the Bicêtre. Some writers lay stress upon the good traits to which warfare gives rise. It would perhaps be more accurate to say that warfare merely emphasises latent tendencies, both of a desirable and an undesirable kind, and that all thus betrayed outwardly by it must already be present *in posse*. The certain effect is that, among its other devastating results, must be reckoned the mental breakdowns and the suicides which are its invariable concomitants. Mental institutions, both public and private, are therefore as much affected in their way as those other medical institutions which exist to treat the physically, as apart from the mentally, wounded. That the mentally wounded are numerous, however little realised by the lay public, is unfortunately not a matter for doubt.

